# Preventing deformities in paediatric cerebral palsy in poorly-resourced areas: A scoping review

**DOI:** 10.4102/sajp.v80i1.2059

**Published:** 2024-10-29

**Authors:** Shayne R. van Aswegen, Mark Richards, Brenda Morrow

**Affiliations:** 1 Department of Paediatrics and Child Health, Faculty of Health Sciences, University of Cape Town, Cape Town, South Africa

**Keywords:** cerebral palsy, child, complications/prevention and control, deformities, low-income setting, parent-implemented

## Abstract

**Background:**

Managing children with cerebral palsy (CP) in poorly-resourced contexts, especially those with greater functional limitations, is challenging. Unmitigated orthopaedic complications can further restrict already compromised functional capacity. Where rehabilitation skills and knowledge are scarce, primary healthcare worker- and caregiver-implemented routines are warranted. The essential elements of a home-based routine to mitigate musculoskeletal (MSK) complications in children with severe CP in resource-limited settings (RLSs) have not been determined.

**Objectives:**

To summarise the evidence for programmes and interventions that mitigate MSK complications in children with severe CP and make recommendations for a programme suited to RLSs.

**Method:**

Scientific databases and professional websites were searched for studies and reports describing guidelines, interventions or programmes for children aged 0–18 years with severe, partially- or non-ambulant CP, that included aims for the prevention of MSK complications. Articles reporting on surgical, pharmacological and advanced or expensive technological interventions were excluded.

**Results:**

A total of 57 studies or reports were included in the review. Low-grade evidence exists for 24-hour postural management (24-h PM), supported standing, sustained stretching and splinting to mitigate MSK complications in children with CP. Caregiver training and support, and integration of the programme into daily routines were identified as important components for successful implementation.

**Conclusion:**

Clinical guidelines and evidence that support caregiver-delivered interventions to reduce MSK complications in children with severe CP are limited, and only weak recommendations can be made.

**Clinical implications:**

There is a need for context-specific, home-based intervention programmes to prevent MSK complications in children with CP in RLSs.

## Introduction

As a leading cause of childhood motor disability worldwide, cerebral palsy (CP) is a complex long-term condition that requires coordinated access to medical interventions, rehabilitation and equipment, all of which are often scarce or unavailable in resource-limited settings (RLSs) (Donald et al. 2015).

**FIGURE 1:**
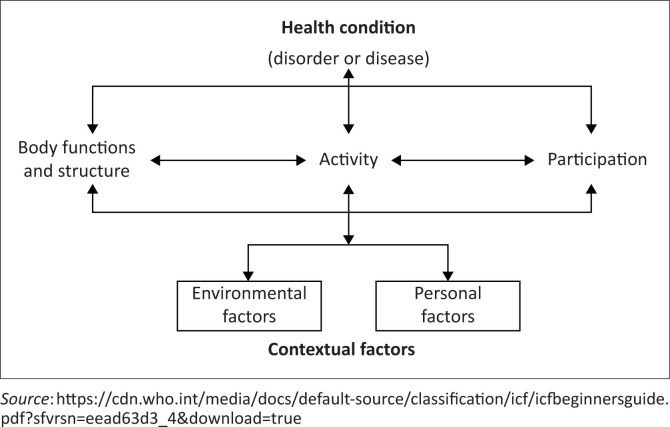
International Classification of Functioning, Disability and Health framework illustrating the interdependence between the various domains.

In low- and middle-income countries (LMIC), the estimated prevalence of CP is between 2 and 10 per 1000 live births, with higher rates in poorer areas (Cans et al. 2008; Couper 2002). Such RLSs likely have higher proportions of the severe types of CP, including bilateral spastic and dyskinetic presentations (Gladstone 2010), which usually occupy the more severe functional levels of the Gross Motor Function Classification System (GMFCS) (Palisano et al. 2007), that is, levels III-V that have no or limited ambulatory ability (Shevell et al. 2009). Children with severe impairments often present with higher rates of co-morbidities, for example, feeding difficulties and orthopaedic complications, of which hip displacement, muscle contractures and scoliosis are common (Hollung et al. 2020). Unmitigated, these can further limit functional status and negatively impact quality of life, as illustrated by the World Health Organization’s (WHO) International Classification of Functioning, Disability and Health framework (ICF) in Figure 1 (WHO 2002).

Using the ICF framework, orthopaedic deformities (Body functions and structure) can prevent the child from being placed in sitting and standing positions, where most functional activities occur (Activity), even where postural devices are available for support. Exclusion from age-appropriate community activities, for example school attendance (Participation), is the likely result, indicating compromised functional health. This population warrants special attention to achieve the United Nations’ (UN) third Sustainable Development Goal (SDG) for 2030, to ‘ensure healthy lives and promote well-being for *all at all ages*’ (UNDESA 2015:6).

Limiting MSK complications is critical to optimise activity and participation opportunities for children with severe CP living in RLSs. Although this requires input from a multi-disciplinary team, specific manual techniques for this purpose traditionally fall within the scope of rehabilitation therapists, which may be a scarce resource in RLSs. Strategies to develop competence in preventing complications within existing rural health structures and within the home are thus warranted.

The value of partnering with primary caregivers to deliver home-based intervention programmes (HBIPs) for chronic conditions is increasingly being recognised even in high-income countries (HICs), where caregivers are trained in the required interventions, then supported and coached by medical professionals (Akhbari Ziegler & Hadders-Algra 2020; Beckers et al. 2020). Adopting this model to manage children with severe CP in RLSs, essential evidence-based interventions aimed at limiting commonly occurring MSK sequelae may be provided by caregivers and/or trained community healthcare workers (CHWs) within the child’s natural environment (ICF domain of Environmental factors). With this aspect adequately addressed, a repertoire of functional activities for the child can be developed (ICF domains of Activity and Participation).

The overall aim of this scoping review was to describe the literature over the last two decades that addresses prevention of MSK complications in children with partially-, or non-ambulant CP (GMFCS levels III-V) that may potentially be used by caregivers in an RLSs, as part of an HBIP.

Specific objectives were to identify:

1. Clinical guidelines for interventions and modalities designed for this purpose and evaluate each recommendation for feasibility in RLSs.
2. Existing HBIPs designed for this purpose and to consider factors affecting programme effectiveness.

## Research methods and design

The broad, exploratory nature of our investigation lent itself to a scoping review, which was guided by the Preferred Reporting Items for a Systematic Review and Meta-analysis extension for Scoping Reviews (PRISMA-ScR)^1^.

Search terms included four concepts: (1) CP in children, (2) musculoskeletal complications, (3) prevention or mitigation and (4) community-based programmes. Preliminary searches revealed little original research in this area during the last decade. Thus, limits were extended retrospectively to 01 January 2001, and searches were run on PubMed, Scopus, Cochrane Library, CINAHL, Health Source: Nursing/Academic Edition, Africa-Wide Information, Web of Science Core Collection and SciELO. Grey literature was identified from Primo, BASE, Clinical Key, EThOS, Google Scholar and Semantic Scholar, as well as professional society websites, associations and bibliographies of relevant publications (Van Aswegen, Richards & Morrow 2024:S1).

Records were imported into the 2020 Covidence web-based collaboration software platform.^2^ One researcher (S.v.A.) screened title and abstracts, and full-text review for eligibility was conducted independently by two researchers (S.v.A. and B.M.), with conflicts resolved by discussion.

### Inclusion criteria

Articles and clinical guidelines with full-text availability, published in the English language between 01 January 2001 and 31 December 2021, that described therapeutic interventions and programmes designed for the prevention of MSK complications for children aged 0–18 years with severe CP (GMFCS levels III–V) were eligible. There was no limit on sample size or study design. Where more than one version of a clinical guideline was retrieved, only the most recent was included.

### Exclusion criteria

- Study population: < 50% related to severe CP diagnosis defined as having limited or no independent ambulation, that is, GMFCS levels III-V; < 50% were 0–18 years of age and studies focussing on unilateral CP, which typically classify as GMFCS level I or II.
- Settings: Interventions requiring specialised environment or expertise not widely available.
- Surgical, radiological and/or pharmacological interventions.
- Outcomes: Not primarily related to prevention or control of MSK complications in CP or HBIPs not including the prevention or control of MSK complications.
- Publication type: Protocols, letters, non-expert reviews, summaries and commentaries and/or editorials.
- Full text of the article is not available.

One researcher (S.v.A.) performed two independent extractions and critical appraisals for each article, at a minimum of 8 weeks apart to improve accuracy and consistency. Study details were then extracted onto a spreadsheet recording first author, year of publication, country or region of origin, sample size, age, diagnosis and GMFCS level, other participants, study design, main subject or concept, intervention, outcomes measured, main results or recommendations, adverse effects and identified gaps in knowledge (Van Aswegen et al. 2024:S2). The level of evidence of each record was noted, based on the work of Ackley et al. (2008), and methodological appraisal was applied using the AGREE-II assessment for clinical guidelines^3^ and Joanna Briggs Institute (JBI) checklists for the other studies.^4^ The JBI checklist and AGREE-II scores were reflected as very low, low, moderate and high, based on percentage of agreement with quality criteria for each record (Van Aswegen et al. 2024:S3, S4, S5).

### Ethical considerations

As this review formed part of a larger study involving human research, ethical approval was obtained for the whole project from the University of Cape Town’s Human Research Ethics Committee with reference no. 024/2022.

## Results

**FIGURE 2:**
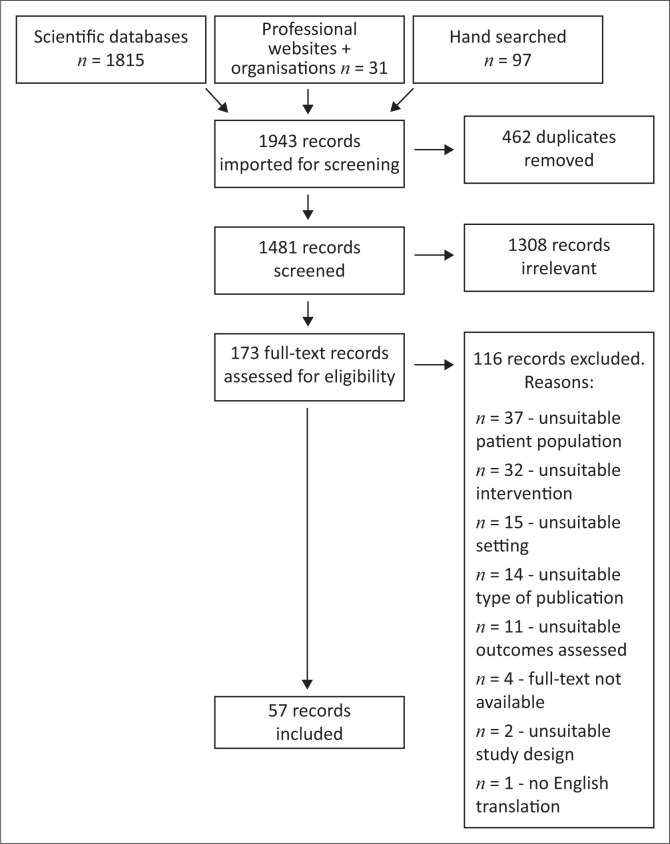
Preferred reporting items for systematic reviews and meta-analyses (PRISMA) flow diagram.

We identified 1943 records. After de-duplication, 1481 records were screened by title and abstract and 173 underwent full-text review. A total of 116 records were excluded as detailed in the PRISMA diagram (Figure 2), which left 57 records for the final review.

The 57 results could be grouped as follows:

1. clinical practice guidelines (CPG) for children with CP (6 articles, 3 of which were systematic reviews [SR])
2. 24-hour postural management (24-h PM), supported seating and standing (27 articles)
3. stretching interventions (12 articles)
4. programmes and aspects of community-based HBIPs from RLSs (12 articles).

Studies in each group were represented chronologically in graphs to illustrate research trends, study type and focus (Van Aswegen et al. 2024:S6).

### Clinical practice guidelines and systematic reviews on clinical recommendations for children with cerebral palsy

Three CPGs came from national or regional health websites in HICs – the National Institute for Health and Care Excellence (NICE) in the United Kingdom (NICE 2012), the Waikato District Health Board (WDHB) in New Zealand (WDHB 2014) and the American Academy for Cerebral Palsy and Developmental Medicine (AACPDM) (Paleg et al. 2019). Two comprehensive SRs of reviews of therapeutic interventions aimed to guide clinical practice (Morgan et al. 2021; Novak et al. 2020), and one article was an SR of guidelines for the care of persons with CP, initiated by the WHO (Damiano et al. 2021). All guidelines adhered to AGREE-II and PRISMA criteria.

The NICE guideline (NICE 2012) and AACPDM Care Pathway (Paleg. et al., 2019) focussed on general medical management of CP and central hypotonia in children. No specific regimens or home programmes were given, but conditional recommendations for interventions targeting the body structures were included. The WDHB guided therapeutic management, providing detailed recommendations for assessment, interventions and equipment needs, all stratified into GMFCS levels and age bands.

One comprehensive SR addressed evidence-based medical and therapeutic interventions for children with CP, but recommendations for children with GMFCS levels IV and V were few and of questionable feasibility in RLSs (Novak et al. 2020).An SR on early intervention guidelines (Morgan et al. 2021) focussed on improving motor function and could make few recommendations in 0- to 2-year-olds for prevention of MSK complications. The WHO review (Damiano et al. 2021) only identified five official guidelines for CP of all ages, and relevant recommendations referenced guidelines already retrieved. Table 1 summarises the recommended non-medical modalities for prevention of MSK complications from each of the included sources.

**TABLE 1:** General recommendations for prevention of mitigate musculoskeletal complications from the retrieved clinical practice guidelines and systematic reviews.

Author, date and study design	Abbreviated title	Population	Recommendations for prevention of MSK complications	Evidence level and quality rating
NICE 2012 Clinical practice guideline	Spasticity in under 19 s: Management	Children with CP, aged 0–18 years	Consider 24-h PM sleeping, sitting and standing with equipment Consider low load stretching Consider ankle and wrist orthoses	Level VII 5/7 Moderate (AGREE-II)
Waikato District Health Board 2014 Clinical practice guideline	CP: Clinical practice guideline	Children with CP, aged 0–18 years	GMFCS IV and V: 24-h PM: Supported standing; seating in a supportive buggy; sleep positioning systems with night or resting splints ankle splints and/or stock boots, gaiters, wrist splints stretches	Level VII 6/7 High (AGREE-II)
Paleg et al. 2019 Clinical practice guideline	Care pathways: Central Hypotonia (CH)	Children with CH (DS, CP and DD), aged 0–6 years	Conditional recommendations: ankle orthotics adaptive equipment, for example, seating and standers 24-h PM	Level VII 4/7 Moderate (AGREE-II)
Novak et al. 2020 Systematic review	State of the Evidence Traffic lights 2019: SR of Interventions for preventing and treating children with CP	247 sources Children with CP, aged 0–18 years	Yellow light – Probably do it: 24-h PM sleep system serial casting Yellow light – Probably don’t do it: stretching orthotics	Level I 8/11 Moderate (JBI)
Morgan et al. 2021 Systematic review	Early Intervention for children aged 0–2 years with or at risk of CP	41 sources Infants at high risk or having CP, aged 0–2 years	Conditional recommendations: use of standing equipment ankle splints do not use a sleeping system	Level I 8/11 Moderate (JBI)
Damiano et al. 2021 Systematic review	SR of Clinical Guidelines related to care of individuals with CP	5 sources People with CP	Consider UL and LL orthoses 24-h PM – standing for BMD Stretching routines	Level I 8/11 Moderate (JBI)

*Source:* Please see full reference list of Van Aswegen, S.R., Richards, M. & Morrow, B., 2024, ‘Preventing deformities in paediatric cerebral palsy in poorly resourced areas: A scoping review’, *South African Journal of Physiotherapy* 80(1), a2059. https://doi.org/10.4102/sajp.v80i1.2059

24-h PM, postural management regime including supported lying, sitting and standing; BMD, bone mineral density, an indication of bone strength; GMFCS, Gross Motor Function Classification System; LL, lower limb; UL, upper limb; CP, cerebral palsy; MSK, musculoskeletal; JBI, Joanna Briggs Institute; CH, Central Hypotonia; DD, developmental delay; DS, Down’s syndrome.

From the results in Table 1, the conditional recommendations for the prevention or control of MSK complications for children with severe CP were:

1. 24-hour postural management, including lying, seating and standing (5 articles); using sleep systems (4 articles, 1 of these implied); supported and/or adapted seating (5 articles, 2 of these implied); standing using relevant equipment (6 articles).
2. Orthotics or splints: For lower limbs (LLs) to prevent loss of range of motion (ROM) in joints and to assist with standing and walking (5 articles) and for upper limbs (ULs) to prevent ROM loss in the wrists (3 articles).
3. Stretching (3 articles).

The next sections discuss the evidence that informs the implementation and application of these recommendations as well as aspects to consider for successful home-based interventions.

### 24-hour postural management, supported seating and standing

At a Mac Keith Multidisciplinary meeting held in 2006, postural management was defined as ‘a planned approach encompassing all activities and interventions which impact on an individual’s posture and function’ (Gericke 2006). For this review, we developed a narrower definition, that is, ‘the supportive positioning of the whole body in lying, sitting and standing to preserve normal MSK alignment and to provide central support for functional activity’. This would include supported standing but exclude a focussed modality such as applying an orthosis.

Of the 27 articles included in this section, all were from HICs, except one from Malaysia (Htwe et al. 2016). Included articles were 18 cohort or quasi-experimental studies (Capati et al. 2020; Gibson et al. 2009; Holmes et al. 2003; Htwe et al. 2016; Kim et al. 2013; Macias-Merlo et al. 2015, 2016; Martinsson & Himmelmann 2011, 2021; Mol et al. 2012; Paleg et al. 2021; Porter et al. 2008; Picciolini et al. 2009, 2016; Pountney et al. 2002, 2009; Tornberg & Lauruschkus 2020; Vekerdy 2007), seven SRs (Chung et al. 2008; Gmelig Meyling et al. 2018; Humphreys et al. 2019; Paleg et al. 2013; Pérez-de la Cruz 2017; Pin 2007; Wynn & Wickham 2009), an expert consensus (Gericke 2006) and a narrative review (Kittelson-Aldred & Hoffman 2017).

Interventions in all three positions were represented in the studies, almost half reporting on standing programmes alone. Outcomes most often related to hip displacement, followed by lower limb ROM. Several sources reported on functional impact of postural management regimens and qualitative measures, for example, ease of performing daily activities, and sleep quality when using sleep systems. Table 2 and Table 3 provide detailed results of studies involving 24-h PM regimes and lying, seating and standing interventions.

**TABLE 2:** Application of postural management: 24 h regimes, lying and seating interventions.

Author, date, design and subject	Population	Intervention and/or exposure	Results and/or recommendations for main outcomes	Evidence level and quality rating
Pountney et al. 2002 Cohort *Effect of a postural management regime on hip displacement*	59 children with CP, aged 0–9.8 years, Unable to sit, had used postural management (PM) for ≥ 2 years including Chailey Adjustable Support systems (CAPS)	SG: ‘All CAPS’ † – (All three modalities) for at least 2 years OR ‘2 CAPS’ (any 2 of the 3 CAPS modalities) OR ‘No CAPS’ (any other system) Mean follow-up 7 years CG: own controls	*Hip migration percentage (MP)* % with both hips safe that is, MP < 33%: - Significant difference in hip status between groups that used ‘All CAPS’ versus groups using ‘2 CAPS’, or ‘No CAPS’ (*p* < 0.05) - ‘All CAPS’ had 65% both hips safe, versus ‘2 CAPS’ with 41.6%, versus ‘No CAPS’ with 12.5%. Should commence by 18 months of age.	Level IV 7/11 Moderate
Holmes et al. 2003 Quasi-experimental *Effect of trunk support position on scoliosis*	16 children and young adults with CP, aged 6.5–20 years, non-ambulant, GMFCS Levels III-V	SG: 3 different configurations of lateral pads to support the trunk in sitting: at pelvis only 2-point arrangement – At pelvis, + lat. pads just under axillae 3-point force arrangement – At pelvis + 2 pads on trunk at different heights CG: own controls	*Thoracic posture* 3-point force system versus 2-point system: - 35% correction in Cobb angle†† versus 18.6%. - Significant ↓ in mean spinous process angles than either configuration 1 or 2 (*p* = 0.000). - Seat force ↓ by 16.8% and a smaller difference in force between trunk pads in configuration 3 (4 N) versus configuration 2 (19 N).	Level III 7/9 High
Gericke 2006 Expert opinion *CPG on PM*	Children with CP, aged 0–18 years	N/A	PM regimes facilitate function, may reduce deformity and can be guided by GMFCS level - GMFCS IV and V should start in sitting from 6 months and standing from 12 months. - GMFCS III should emphasise postural activity.	Level VII 5/6 High
Vekerdy 2007 Quasi-experimental *Effect of seating device on spinal posture and daily activities*	47 children with CP, aged 1–11 years, GMFCS III-V	SG: Sitting in a custom-moulded trunk support (TLSO with SIDO^®^ frame) ‡ for an average of 3.9 h per day for at least 4 months Mean follow-up of 12.7 months CG: own controls	*Seated posture* Most improved with ↓ thoracic kyphosis (*p* < 0.0001) and ↑ lordosis (*p* = 0.0025). - Cobb angle†† ↓ but not significant. *Daily activities (according to parents):* - Feeding problems ↓ in 91.4% of children. - Trunk posture improved in 88.65%. - Average satisfaction with device – 3.9/5.	Level III 6/9 Moderate
Chung et al. 2008 Systematic review *Effect of adaptive seating on sitting posture and postural control*	14 sources, mostly moderate quality > 176 children and young adults with CP, varying GMFCS levels	Using various approaches to improve seating posture: CAPS II§, seat tilt, seat inserts, knee blocks, saddle seats, 2-point and 3-point support, modular seating (pelvic symmetry, seat sloped forward, hips in Abd. feet supported), tray tables and child’s own chair	Evidence is limited and strong recommendations not possible. *Sitting posture for moderate to severe CP* - Saddle position: Mixed results. - Seat tilt: No tilt and a 5° posterior tilt ↑ stability (*p* ≤ 0.05) - Seat inserts: Contoured versus flat foam had significant positive effect on maintaining posture over time (*p* = 0.008) and biofeedback devices ↑ postural stability subjectively. - 3-point trunk support versus 2-point significant ↓ in scoliosis (*p* = 0.000). - Modular seating versus regular seating (e.g. standard wheelchair) improved spinal posture (*p* ≤ 0.001). *Social interaction:* Subjective improvements in social skills, ADL independence and feeding.	Level II 8/11 Moderate
Porter et al. 2008 Cohort *Effect of preferred positioning on hips and spine*	246 children with CP, aged 0–18 years, median aged 10 years 3 months, GMFCS level V	SG: Asymmetrical holding and feeding positioning and preferred lying posture during first 12 months of life CG: own controls	*Musculoskeletal (MSK) deformities* - 95.5% had lateral curve in their spine, 82.5% had pelvic obliquity, 60.2% had windswept posture and 48.8% at least one displaced hip. - When preferentially using side lying: 69.0% used one side only. - When using supine and prone: > 50.0% consistently rotated head to one side. - Side lying on a preferred side associated with a spinal curve convex to the top hip (*p* = 0.031) and more likely to experience hip problems (displacement) in the top hip (*p* = 0.003).	Level IV 6/11 Moderate
Pountney et al. 2009 Cohort *Effect of PM on hip displacement*	39 children with CP, aged 18 months to 5 years, non-ambulant CG: 202	SG: early use of ‘All CAPS’† PM programmes or ‘2 CAPS’ moderate use (2 modalities × 6 h per day) daily CG: minimal use	*Hip outcomes* - MP < 33%: SG had 59% both hips ‘safe’ versus 50% in CG. - % hip problems requiring surgery, BoNT or a hip and spinal orthosis at 5 years: SG was 18.2% less than for CG (*p* = 0.006). - Hip surgery: SG significantly less likely than CG (*p* ≤ 0.001).	Level IV 7/11 Moderate
Picciolini et al. 2009 Case series *Effect of PM on hip displacement*	2 children with CP, aged 0–5 years	5 h per day seated and standing in a customised hip Abd. cast (siège moulé and gouttière)¶ for at least 2.5 years. CG: own controls	*Hip displacement* - First case: from 2.5 to 5 years of age; seating resulted in restoration of pelvic symmetry, bilateral MP of 20% and no scoliosis. - Second case: started using siège moulé and gouttière 5 h per day at age 7; at age 10 years, MP right ↓ from 55% to 16% and MP left from 20% to 15%.	Level VI 5/10 Low
Wynn and Wickham 2009 Systematic review *Effects of SS on children and families*	6 sources, mostly of low quality > 100 participants of families and children with CP or ‘postural care needs’	Using a sleep system every night for a varying period of 6 months to several years	Very small body of evidence. *Parental opinion on daily care* - After 1 year, > 50% had better symmetry, ↓ tone and pain, care was easier. - Majority of parents required training in use. *Hip MP* - Significantly ↓ after 6 months of using sleep systems (*p* < 0.05).	Level V 4/11 Low
Mol et al. 2012 Analytical cross section *Effect of night orthoses on sleep and burden of care*	82 children with CP, aged 6–15 years	Questionnaires filled out by primary caregiver and physiotherapists Domains: Sleep disturbance; parental burden, parental personality and sense of competence	*Sleep disturbance in CP* - Prevalence of 20.7%, most using some type of sleep orthosis; however, no significant difference found between those using night orthoses and not (*p* = 0.28) or between those using them day and night, day only or night only (*p* = 0.25). - Daytime use only had significant disturbance in initiating and maintaining sleep (*p* < 0.05) compared to day and night use or night use only. *Burden of care* - Parental extraversion negatively correlated with experienced burden and positively with sense of competence.	Level VI 6/8 Moderate
Kim et al. 2013 Cohort *Effect of moulded chair on hip and spine*	34 children and young adults with severe disability, aged 4–20 years, GMFCS level V	SG: Use of a custom-moulded chair for an average of 3.7 h per day for an average of 24 months CG: own controls	*Hip and spine outcomes* - Femur neck shaft angle‡‡ – significant ↓ in 10 to 15-year-olds ‘growth spurt group’ but group was very small (*p* < 0.05). - Cobb angle†† and MP: No significant difference but hips did not worsen over the period.	Level IV 6/11 Moderate
Picciolini et al. 2016 Quasi-experimental *Effect of Abd. seating on hip displacement*	51 children with CP, aged 6 months – 9 years, GMFCS III-V	SG: 5 h per day seated in the siège moulé¶ + neurodevelopmental therapy for 2 years. CG: only neurodevelopmental therapy	*Hip displacement* Hip MP in SG ↓ from 28% to 26% showing a small improvement, while CG worsened significantly over the 2 years from 23% to 37% (*p* ≤ 0.0001).	Level III 6/9 Moderate
Kittelson-Aldred and Hoffman 2017 Narrative review *Evidence summary on PM*	Persons requiring postural support	23 sources	Postural asymmetry is very common, and even obligatory in children with impaired movement especially as they age. - In spite of lack of evidence, 24 h PM is widely accepted and used. Most effective when applied over 24 h and as an interdisciplinary approach between healthcare professionals, parents and individuals.	Level VI 4/6 Moderate
Pérez-de la Cruz 2017 Systematic review *Effects of PM on hip displacement*	18 sources children with CP, aged 18 months – 18 years	Varying use of CAPS† systems: a static Abducted sleep system, Abducted sitting and standing using a siège moulé and gouttière¶	*Hip displacement* Evidence insufficient for strong recommendations - Hip MP: 24 h PM using ‘All CAPS’ or ‘2 CAPS’, MP was significantly ↓ after 2 years. - Early intervention using PM resulted in significant ↓ in surgery and BoNT after 5 years. - Children using a static abduction sleep system for 18 months had 11% ↓ in MP, improved hip ROM and gait pattern. However, 50% found sleep systems uncomfortable. - Children using abducted sitting and standing for 5 h p per day for 3 years had a significant ↓ in MP.	Level III 3/11 Low
Gmelig Meyling et al. 2018 Systematic review *Effects of PM on hip migration*	8 sources, mostly of low-quality Children with CP, aged 0–18 years, GMFCS levels III–V	SG: Sleep system every night, siège moulé¶ or Abducted sitting from 1 to6 h per day; Abducted or straddled standing 1–5 h per day Duration 1–7 years. CG: received usual care (Neurodevelopmental therapy 45 min 3 × per week)	Strong recommendation not possible. *Hip outcomes* - Siège moulé users had significant ↓ in MP after 2 years (*p* = 0.03). - Abducted standing/straddled weight bearing had a significant ↓ in MP (*p* < 0.01 and *p* = 0.029). - Children using full 24 h PM maintained a stable MP significantly more than CG (*p* = 0.05). Frequency of hip problems in SG versus CG: - PM in lying, sitting and standing had significantly ↓ at 5 years (*p* < 0.01). Frequency of children requiring surgery, orthotics and BoNT was significantly ↓ in the SG at 5 years (*p* ≤ 0.01).	Level III 9/11 High
Humphreys et al. 2019 Systematic review *Effect of night orthoses on hips, sleep and burden of care*	People with a neuro-disability	14 sources of low to medium quality Sleep systems all night for 8 days – 18 months using commercially available sleep systems – Jenx Dreama, Symmetrisleep, Chailey lying support or unspecified	Weak evidence, therefore strong inferences of benefit not possible *Hip outcomes* - Pain and comfort: ↓ pain in some children but ↓ thermal comfort. - Hip MP: Significant ↓ in MP on the ‘worse’ hip after 12 months, and 92% MP stability or improvement after 12 months. - ROM: Hip abduction improved in 66% of patients (small samples and low-quality evidence). *Sleep quality, burden of care* - No evidence that sleep systems ↓ sleep quality, rather, they sometimes ↓ nocturnal awakenings. - Respiratory function: No significant difference reported between users and non-users. - Quality of life and burden of care: Weak evidence that sleep systems have positive influence. - Parent training and adherence: 20% patients have difficulty adapting to sleep systems (vomiting, reflux and discomfort): Parents felt they needed support while children adapted.	Level II 7/11 Moderate

*Source:* Please see full reference list of Van Aswegen, S.R., Richards, M. & Morrow, B., 2024, ‘Preventing deformities in paediatric cerebral palsy in poorly resourced areas: A scoping review’, *South African Journal of Physiotherapy* 80(1), a2059. https://doi.org/10.4102/sajp.v80i1.2059

Note: Abd., Abduction or abducted (hips), lateral movement in frontal plane away from the midline; Early intervention, applied to children younger than 2 years; Hips ‘safe’, having lateral displacement less than 33%; Kyphosis, curvature of the spine in the sagittal plane away from the central axis (‘hunch back’); Lordosis, curvature of the spine in the sagittal plane towards the central axis (‘sway back’); MP, hip migration %, indicating lateral hip displacement – safe hip has MP < 33%, > 33% the hip is subluxed and at 80% the hip is dislocated; Neurodevelopmental therapy, widely accepted manual approach for habilitation of CP that aims to promote efficient movement and optimise function; Pelvic Obliquity, rotation of the hips in the frontal plane, resulting in one iliac crest presenting higher than the other; Saddle position, hips in flexion and abduction, similar to Siège moulé; Scoliosis, lateral curvature of the spine in the frontal plane away from the central axis; Windswept posture, multiple primary and compensatory lateral and rotational deviations of spine, hips and lower limbs from the midline.

ADL, activities of daily living (incl. bathing, feeding dressing, toileting); BoNT, Botulinum Neurotoxin injection; CG, control group; CP, cerebral palsy; CPG, clinical practice guidelines; GMFCS, Gross Motor Function Classification System; lat., lateral; MSK, musculoskeletal; N, Newtons; N/A, not applicable; PM, postural management; QoL, quality of life; ROM, range of motion; SG, study group.

† , CAPS, Chailey Adjustable Postural Support Systems. ‘All CAPS’ involving full sleep system at night, 6 h abducted sitting per day, 1 h standing per day.

‡ , TLSO with SIDO^®^, Thoraco-lumbar-sacral-orthosis, a back brace with a flexible support.

§ , CAPS II, modular positioning system (manufactured by Active Design, Ltd 2016) that allows fully customised postural support of the body.

¶ , Siège moulé and Gouttière, customised plaster cast seating and standing supports, respectively.

†† , Cobb angle, radiological measurement of lateral spinal curvature for scoliosis.

‡‡ , Femur neck shaft angle, angle between femur neck and shaft. Normal hips 125° – 135°; > 140° is associated with hip displacement.

**TABLE 3:** Application of postural management: Supported standing interventions.

Author, date, design and subject	Population	Intervention and/or exposure	Results and/or recommendations	Quality – JBI checklist
Pin 2007 Systematic review *Effects of static WB on MSK systems*	10 sources, > 100 children with CP, aged 17 months–14 years, Various GMFCS levels	Weightbearing (WB) in UL and LL, in various positions and various regimes and dosages ranging from 4 weeks to 9 months.	Limited strength of recommendations for all outcomes, except for bone mineral density (BMD) where there is strong evidence. *MSK outcomes after static WB in LL* - BMD ↑ significantly with 20 min WB, 3 × per week for 8 months (*p* = 0.02). - Gait: Standing in a tilt table for 30 min led to a significant improvement in gait (*p* < 0.01). - Spasticity: ↓ in resistance to passive movement for up to 35 min (*p* < 0.05). Benefits of WB for UL is inconclusive.	Level II 8/11 Moderate
Gibson et al. 2009 Quasi-experimental *Effects of standing programs on LL deformities*	5 children with CP, aged 5–10 years, GMFCS IV and V	SG: 2 × Intervention periods of 60 min standing in a frame per day, 5 days per week for 6 weeks, alternating with 2 × non-intervention periods (no standing) for 6 weeks CG: own controls	*Knee ROM* - Improved significantly during the 2 intervention phases (*p* < 0.01 and *p* = 0.03). - ↓ significantly during first non-standing phase (*p* = 0.02). *Ease of performing ADLs*† - ↑ after standing phases (mean scores > 0.0) and a small ↓ following non-standing phases (mean scores < 0.0).	Level III 7/9 High
Martinsson and Himmelmann 2011 Quasi-experimental *Effect of WB in Abduction on hip stability*	97 young children with CP, aged 2–6 years, GMFCS III–V	SG: 60–90 min per day in straddled standing (with hip Abd. of 15° – 30°) for 1 year CG: usual standing programme without hip Abd. Both SG and CG had 2 subgroups: those who had hip release surgery (SG1 and CG1) and those who had not (SG2 and CG2).	*Hip MP* - Significant correlation between MP and straddled standing (SG1 and SG2) with a mean ↓ of 8.6% (*p* = 0.000). - Significant correlation between having hip surgery (SG1 and CG1) and MP, with a mean ↓ of 11.9% (*p* = 0.000). - In SG1 (surgery followed by straddled standing) mean ↓ in MP was 20.8% (*p* = 0.035). *Hip and knee ROM* - No significant difference between groups for hip and knee Ex. - Hip Abd. improved significantly by a mean of 15° with straddled standing between SG1 and CG1 (*p* = 0.002).	Level III 6/9 Moderate
Paleg et al. 2013 Systematic review *Dosing of paediatric supported standing programs*	30 sources and > 100 young children with CP, aged 1–6 years, GMFCS II–V	Various regimes and standing devices. 30–90 min, 3–7 days per week, with 0° – 70° hip Abd. Duration from 3 weeks up to 1 year.	Summary and recommendations for all outcomes: - For ↑ ROM in LL: standing with Abd. 45–60 min per day from 9 months of age, 3 days per week (strong evidence). - For ↑ muscle strength: 5 days per week for 10 min twice per day with WBV may ↑ muscle power (good evidence). - For ↓ muscle tone: 30–45 min standing per day 3 days per week (strong evidence). - For ↑ hip stability (↓MP): 30–90 min per day in 60° Abd., 5–7 days per week may improve hip biomechanics (fair evidence). - For ↑ BMD of spinal vertebrae: ↑ by 6% after 30–90 min per day, 5 days per week; when combined with WBV‡, BMD in tibia ↑ of 18%. - For ↑ GM function: standing may ↓ feeding times, promote social interaction and ease burden of care.	Level I 8/11 Moderate
Macias-Merlo et al. 2015 Cohort *Effect of standing programs on hip flexibility*	13 young children with diplegic CP, aged 1–5 years, GMFCS III	SG: 70–90 min in a stander with hip Abd. 5 days per week, 35 min per day on weekends until the age of 5 years CG: own control	*Hip flexibility (ROM)* Mean hip Abd.: At baseline was 42.0° (95% CI 41.0° – 43.0°) and at age 5 years was 42.8° (95% CI 41.8° – 43.8°) - Reduction in scissor gait.	Level IV 6/11 Moderate
Macias-Merlo et al. 2016 Cohort *Effect of standing programs on hip MP (acetabulum development)*	26 young children with diplegic CP, aged 1–5 years, GMFCS III	SG: 70–90 min in a stander with Abd. 5 days per week, 35 min on weekends until the age of 5 years CG: matched but did not participate in standing.	*Hip MP and symmetry* For the worst hip: maximum MP in SG was significantly ↓ at 5 years – 20.23% (s.d. 2.42) versus CG at 5 years – 35.15% (s.d. 7.30) (*p* = 0.000). - Mean MP difference: Standing group 4.00% s.d. 2.74 versus CG 18.31%, s.d. 7.52 (*p* < 0.05). - Hip asymmetry: Ranged from 13% – 23% in SG versus 12% – 47% in CG, which is significant (*p* < 0.01).	Level IV 7/11 Moderate
Htwe et al. 2016 Analytical cross section *Effects of standing programmes on hip displacement*	36 children with CP, aged 6–12 years, GMFCS III to V	Intervention and results not well-documented. Various standing regimes – for between 1 h-3 h per day, 5 × per week for a duration of 4-10 years.	*Hip MP and acetabular index (AI)*§ - The higher the GMFCS level, the greater the MP and AI§ values (*p* < 0.05). - MP of GMFCS IV and V were 32.5% and 30.0%, respectively, in children who had been standing for at least 4 years for at least 1 h per day at least 5 days per week but also having regular PM and regular physiotherapy.	Level VI 4/8 Low
Capati et al. 2020 Case report *Effect of standing programme on hip and knee ROM*	Single subject, adolescent with CP, aged 16 years, GMFCS V	60 min 3 × per week for 15 months in a stander using progressively increasing joint Ex and reducing stander inclination until fully upright Own control	Hip and knee ROM - Hip F contractures ↓ from 25°to 0° on right and from 40° to 20° on left. - Knee F contractures ↓ from 40 to 20° on right but ↑ from 30° to 35° on left. *Function and ease of care* - In participation and activity levels, no change. - Ease of care: at 7- and 15-month post intervention, reported greater ease with bathing, dressing and transferring and with bowel care.	Level VI 7/8 High
Tornberg and Lauruschkus 2020 Quasi-experimental *Effect of static vs dynamic standing on LL ROM and muscle tone*	20 children with CP aged 0–18 years, non-ambulatory (GMFCS IV and V)	SG and CG crossover: 4-month trial: During static standing phase: Standard care + static standing 30–90 min per day. During dynamic standing phase: 30 min per day at 30–50 revs per min in the Innowalk apparatus.¶	*Hip and knee ROM* - Pre- and post-intervention ROM improved significantly with both static and dynamic standing (*p* = 0.001). - Difference in ROM changes after each session was significantly larger after dynamic versus static standing for all movements (all *p* ≤ 0.003). *Spasticity* - Acute ↓ after sessions of dynamic standing were significant for all hip movements (Abduction, Flexion and Extension) (*p* values all < 0.001) versus static standing where only hip Flexion was significantly improved (*p* = 0.04). - No significant long-term difference in tone was found between dynamic and static standing pre-and post-intervention.	Level III 7/9 High
Martinsson and Himmelmann 2021 Cohort *Effects of Abd. standing on hip development and LL ROM*	269 children with CP enrolled in CPUP before 2 years, aged 3–16 years, GMFCS IV and V	SG and CG: Use of a stander/standing shell 10 h per week for at least 8 months with SG: 15° hip Abd. or more CG: Same use of stander with hip Abd of 0° – 10°.	*Hip MP* - Mean ↓ in MP with SG was 12% and a sig. correlation between Abd. standing and change in MP (*p* = 0.001). - Median MP between SG ↓ by 7% versus CG where MP ↑ increased by 6.5% (*p* = 0.001) indicating a s.d. *Hip and knee ROM* - New contracture development in SG versus CG was 0 joints and 41 more joints respectively. - With those undergoing AIT surgery†† prior to intervention, significant ↑ in ROM of hip Abd. and knee Ex were found (*p* = 0.013 and *p* = 0.04, respectively).	Level IV 10/11 High
Paleg et al. 2021 Analytical cross section *Effect of inclination hip Abd. and orientation on WB in standing devices*	15 children with CP and other conditions (2 typically developing), aged 3–9 years, GMFCS III–V	Standing in a total of 36 positions of varying inclination, orientation and hip Abd.	*Effect of standing position on WB* - For hypertonus (spasticity) in limbs: Max WB (91%) occurred in prone position, upright, hips in neutral or slight Abd. - For hypotonus (low tone) in limbs: max WB (95%) in supine orientation, upright, hip Abd. of 60%.	Level VI 6/8 Moderate

*Source:* Please see full reference list of Van Aswegen, S.R., Richards, M. & Morrow, B., 2024, ‘Preventing deformities in paediatric cerebral palsy in poorly resourced areas: A scoping review’, *South African Journal of Physiotherapy* 80(1), a2059. https://doi.org/10.4102/sajp.v80i1.2059

Note: Abd., Abduction or abducted (hips); Lateral movement in frontal plane away from the midline; MP, hip migration percentage, indicating lateral hip displacement – safe hip has MP < 33%, > 33% the hip is subluxed and at 80%, the hip is dislocated; Scissor gait, legs cross the midline during ambulation because of hypertonus in the hip abductors; spasticity (or hypertonus), increase in muscle tone in response to a quick stretch. It is caused by central hypertonia; Tilt table, supported standing device that allows inclination from the vertical position.

Abd., Abduction or abducted (hips); BMD, bone mineral density (strength); CG, control group; CP, cerebral palsy; CPUP, national cerebral palsy hip surveillance programme; Ex, extension; GM, gross motor; GMFCS, Gross Motor Function Classification System; JBI, Joanna Briggs Institute; LL, lower limb; MSK, musculoskeletal; ROM, range of motion of a joint (flexibility); sig., significant; SG, study group; UL, upper limb; WB, weight bearing, usually with respect to standing.

† , ADL, activities of daily living (incl. bathing, feeding dressing, toileting);

‡ , WBV, whole body vibration performed by a device (stepping, oscillations, vibrations);

§ , AI, acetabular index, a radiological measurement of hip inclination;

¶ , Innowalk apparatus, a dynamic supported standing device that allows hip movements that approximate walking;

†† , AIT, abductor ileo-psoas tenotomy (surgical soft tissue release of inner hip muscles).

For children in GMFCS levels IV and V, the expert consensus from 2006 recommended lying or sleeping with postural support at night, sitting from 6 months of age and supported standing from 12 months (Gericke 2006) The researchers found no revisions or updates for this regimen.

Unsupported holding, feeding and lying positions for children with GMFCS level V tended to result in spinal, pelvic and hip deformities in up to 95% of subjects (Porter et al. 2008). However, using a consistent 24-h PM approach in lying, seating with hip abduction for 5 to 6 h daily (Kim et al. 2013) and/or standing from the age of 18 months and upwards for 30–90 min per day significantly controlled and/or improved hip migration percentage (MP) and reduced frequency of spinal orthotic prescription, Botulinum toxin injections (BoNT) and hip surgery indications at age 5 (Gmelig Meyling et al. 2018; Pérez-de la Cruz 2017; Picciolini et al. 2016, 2009; Pountney et al. 2002, 2009).

Weak and inconclusive evidence from three studies reported the effects of using commercial sleep systems. Two reported significant reduction of hip MP after a minimum of 6 months and up to several years of consistent use, and hip ROM improved along with MSK comfort levels. Thermal comfort, however, was sometimes reduced, and parents required training to use these systems (Humphreys et al. 2019; Wynn & Wickham 2009). Caregivers reported that sleeping systems did not cause significant sleep disturbance, especially if used night and day (Mol et al. 2012).

Seating that incorporated a 3-point force system (Holmes et al. 2003), or modular or moulded contours with optional tilt, produced a decreased spinal Cobb angle and improved thoracic posture when used for at least 4 h per day (Chung et al. 2008). In addition, caregivers from two studies found trunk-supported seating eased everyday caregiving and improved the child’s social interaction (Chung et al. 2008; Vekerdy 2007).

Supported standing regimes were reported to significantly improve a range of outcomes, especially when commenced as early as 9 months of age (Paleg et al. 2013). Standing for 30 to 90 min per day for 3 to 7 days per week for young children from the age of 12 months up to 5 years significantly decreased hip MP at 5 years of age, the effects enhanced with additional total hip abduction up to 60° (Htwe et al. 2016; Macias-Merlo et al. 2016; Martinsson & Himmelmann 2011, 2021; Paleg et al. 2013). Standing for at least 30 min per day for at least 3 days per week also improved bone mineral density (BMD) significantly in the LLs and spine, especially when combined with whole body vibration (Paleg et al. 2013; Pin 2007) and reduced spasticity in the LLs for up to 35 min post intervention, which in turn led to a less scissored gait pattern (Macias-Merlo et al. 2015; Paleg et al. 2013; Pin 2007; Tornberg & Lauruschkus 2020). Standing for 45-60 min per day 3-5 days per week for 8-15 months was found to significantly increase LL ROM, which led to improvements in gait, social interaction and decreased burden of care (Capati et al. 2020; Gibson et al. 2009; Macias-Merlo et al. 2015; Martinsson & Himmelmann 2021; Paleg et al. 2013; Tornberg & Lauruschkus 2020).

### Stretching and splinting interventions

Twelve articles, mostly from HICs, were included in this section, but only three were primary studies – a case-controlled time series from 2003 (Fragala et al. 2003) and two quasi-experimental trials (Laessker-Alkema & Eek 2016; Uzun Akkaya & Elbasan, 2021). The rest included six SRs (Autti-Rämö et al. 2006; Craig et al. 2016; Eldridge & Lavin 2016; Groppe et al. 2012; Wiart et al. 2008), two narrative reviews (Physiopedia 2016; Wilton 2003) and an expert consensus (Lannin et al. 2011). Within the five SRs that included manual stretching, there was considerable overlap in the sources, with several studies repeatedly cited (Craig et al. 2016; Eldridge & Lavin 2016; Groppe et al. 2012; Pin et al. 2006; Wiart et al. 2008). Overall, the evidence was scarce.

Although stretching is widely regarded as an essential part of conservative joint range maintenance in CP (Wiart et al. 2008), the researchers found no clear consensus on the scope of ‘stretching’ techniques nor any standardised stretching dosage regimes. All sources described manual (intermittent) stretching of a joint or muscle by a therapist, maintained for up to 60 s per repetition. Most sources also included at least one of the following: manual stretching with the addition of electrical stimulation (ES); active stretching with child participation; positional or sustained stretching, lasting 15 min or more and using equipment such as a standing frame or an orthosis; intramuscular stretching (similar to massage) and serial casting where a series of rigid casts were applied to a joint for progressive lengthening of shortened tissues. As the use of orthotics in children with severe CP is largely for joint range maintenance (Lannin et al. 2011), five studies reporting on orthotic use were included here. Outcomes included calf muscle spasticity, LL ROM and gross motor function. Characteristics of studies on stretching interventions are presented in Table 4.

**TABLE 4:** Effects of stretching interventions for children with severe cerebral palsy.

Author, date, design and subject	Population	Intervention and/or exposure	Results and/or recommendations	Quality
Fragala et al. 2003 Case-controlled time series *Effects of regular stretch routine on LL ROM*	7 children with CP, aged 4–18 years, Severe mobility limitations (GMFCS likely III–V)	Intervention phase: Usual care = manual stretches 3 × 60 s of hip, knee and ankle joints, 1–2 × per week + classroom positioning for the duration of school term. 2x non-intervention phases during holidays of 5 weeks and then 3 weeks. Own controls.	*ROM (hip flexion, extension abduction and knee flexion and extension)* After first non-intervention of 5 weeks – significant ↓ in LL ROM in 7 subjects (*p* = 0.046) but change after 3 weeks non-intervention was not significant.	Level IV 8/11 Moderate
Wilton 2003 Narrative review *Effects of orthotics on hand deformities and function*	Children with CP	Casting, splinting and stretching of the hand.	*Hand ROM* - Stretch is essential to muscle growth and to maintain length after growing stops. - Casting 24 h per day for 4 weeks ↑ ROM. - Intermittent casting for 3–5 h per day using splints and casts is best for effective stretch in growing muscles with moderate to severe spasticity. - Night splinting to address hand deformities and control the thumb is recommended. - For spastic muscles, contractures recur unless stretch routine is maintained. *Hand function* Improved ROM and addressing deformities do not necessarily improve function.	Level VII 4/6 Moderate
Autti-Rämö et al. 2006 Systematic review *Summarise effectiveness of UL and LL casting and orthoses on ROM and function*	5 sources, > 600 children with CP, GMFCS varied	Any intervention using casts or orthoses for ROM or function in LLs and ULs, dosages and regimes not specified.	Results mostly inconclusive *ROM* - LL orthoses – no evidence to support prevention of ankle deformity; moderate evidence that casting increased ROM in the short term. - UL orthoses – no evidence that they increase or maintain ROM. Casting as an adjunct to therapy for 4 weeks – 6 months ↑ ROM. *Function* - LL: ankle movement improved during gait. - UL orthoses may improve grasp but may restrict activity.	Level I 8/11 Moderate
Pin et al. 2006 Systematic review *Effectiveness of passive (manual) stretch on ROM and spasticity*	7 sources, > 100 children and young adults with CP, aged 3–20 years, various GMFCS levels	Various: Manual stretch of varying dose and duration; standing programmes for 30 min, variable duration and frequency.	Conflicting evidence for manual stretching Weak evidence that sustained stretch may be more effective than manual stretching to ↑ ankle movement and ↓ spasticity. *Spasticity* - 30 min sustained stretch 3 × per week × 6 weeks showed a significant ↓ (*p* < 0.01). - 30 min standing in tilt table led to significant ↓ (*p* < 0.05).	Level II 8/11 Moderate
Wiart et al. 2008 Systematic review *Effect of stretching on ROM, spasticity, muscle activation and gait*	7 sources, > 100 children with CP, aged 3–13 years, GMFCS levels varied	Various: Manual stretching of various dosages and duration; standing programmes for 30 min with variable duration.	Insufficient evidence to support any stretch interventions, despite widespread use. *Hip and knee ROM and spasticity* Inconsistent, insufficient results. *Muscle activation and gait* No evidence to support.	Level III 6/11 Moderate
Lannin et al. 2011 Expert opinion *CPGs for orthotic management of CP*	People with CP	Orthotics and effects on deformities, gait and general health.	*Recommendations* In GMFCS levels IV and V orthoses may help prevent LL joint deformities and provide a prolonged stretch to maintain muscle length: - Hip orthoses have poor patient adherence. - Spinal orthoses may slow the rate of scoliosis. - UL orthoses have inconclusive evidence for improving ROM. - The use of UL orthoses may hinder function.	Level VII 5/6 High
Groppe et al. 2012 Systematic review *Effect of stretching on ROM and spasticity*	13 sources, > 150 children with CP, aged 2–19 years, GMFCS various	Manual stretch: - 3 × 30–60 s 1–5 days per week, one study adding electrical stimulation (ES) of antagonist for 30 min 3 × per week applied to LLs for up to 5 months. - sustained positional stretching (sitting with legs in hip abduction and knee extension for 20 min). - serial casts for 3-12 weeks, - standing for 30 min applied to LLs.	Weak evidence that stretching improves MSK or functional outcomes. *Hip and knee ROM* - Manual stretching + ES led to significant ↑ in knee Ex (*p* = 0.04) in a single study. - Positional stretching in sitting led to significant mean ↑ in ankle DF (9.25°). - serial casting for 3 weeks ↑ ROM LL (all *p* < 0.001). *Spasticity* - Manual stretching + ES led to significant ↓ (*p* = 0.046) in a single study. - Positional stretching in sitting for 20 min led to significant ↓ in Hoffman reflex† of 5.17mV (s.d. 3.61). - 3 × 30 min standing per week for 5 months led to significant ↓, on average by 50% for up to 35 min post intervention (*p* ≤ 0.05). - Serial casting for 3 weeks – all participants had significant ↓ using various measures.	Level III 6/11 Moderate
Craig et al. 2016 Systematic review *Effectiveness of stretch interventions on ROM, BMD, spasticity and GM function*	16 sources, > 4000 children with neuromuscular disabilities; age and level unspecified and various	Any stretch technique (manual stretching bracing, splinting and orthoses, positioning programmes, casting) applied to body structure, function, activity, or participation.	Limited evidence for benefit to body structures. *ROM and spasticity* - Manual stretching: Insufficient and conflicting evidence for efficacy. - Sustained stretch (positional or with orthosis for at least 30 min) appears more effective than intermittent passive stretching (low evidence). - Orthotics had conflicting and insufficient evidence for efficacy. - Supported standing of 30–90 min per day: ↑ BMD (low-quality evidence) but for ROM and spasticity had insufficient evidence. - Casting: ↑ROM in ankle in short term. - Splinting may cause bruising, skin breakdown or sleep disturbance.	Level II 8/11 Moderate
Eldridge and Lavin 2016 Systematic review *Effect of stretching on ROM and spasticity of the calf muscle*	4 sources, unreported sample size, children with CP, aged 3–14 years, GMFCS level unspecified or varied	Manual stretch of the gastrocnemius 5 × 20 s (calf) performed by HCP versus client self-stretch and 30 min supported standing.	Very weak, conflicting evidence that stretching influences ROM and tone. *ROM (one study)* Passive stretching: A transient significant ↑ in both ROM and soft tissue length (all *p* < 0.001) whether performed by HCP or client. *Calf spasticity* Contradicting evidence; the study that observed a significant ↓ in tone found that the effects remained after 35 min (*p* < 0.05).	Level III 6/11 Moderate
Laessker-Alkema and Eek 2016 Quasi-experimental trial *Effect of knee orthotics on ROM, spasticity and GM function*	10 children with CP, aged 1–15 years, all GMFCS levels	Application of splints at least 30 min per day for at least 5 days per week for 8 weeks with knee-ankle-foot orthoses (KAFO) in maximum knee extension (Ex). Own controls.	*ROM* Sustained stretch: Hamstring extensibility significant ↑ compared to baseline (*p* = 0.005 for both L and R) Knee Ex: Significant ↑ (*p* = 0.028 on R, *p* = 0.018 on L). *Spasticity* Hamstrings: Significant ↓ in half of the children on both sides (*p* = 0.005). *Function and ease of care* No change in GM function, but parents and teachers reported that ADLs became easier, and half of the parents intended to continue with the intervention after the research. Adverse effects: Knee swelling, muscle cramps.	Level III 6/9 Moderate
Physiopedia editors 2016 Narrative review *Orthotic management of CP*	People with CP	Various types and uses of orthoses, dosage unspecified.	*ROM, posture and spasticity* - For GMFCS IV-V, hip Abduction orthotics don’t prevent hip displacement but may improve sitting posture and symmetry. - Ankle splints manage spasticity and maintain ROM for standing. - Thoraco-lumbar-sacral orthotics may retard or prevent scoliosis progression.	Level VII 3/6 Low
Uzun Akkaya and Elbasan 2021 Quasi-experimental trial *Acute effects of stretching on spasticity of calf muscles*	22 children with CP, aged 5–13 years, GMFCS levels III-V	10 min of intramuscular stretching on one calf muscle and 10 min of intermittent 15 s passive stretch:15 s rest on the other side. Own controls.	Weak evidence that manual stretching decreases spasticity. *Spasticity* Both methods → significant ↓ spasticity in the acute phase using MAS‡ (*p* = 0.024): - Difference between the two methods not significant (*p* > 0.05).	Level III 6/9 Moderate

*Source:* Please see full reference list of Van Aswegen, S.R., Richards, M. & Morrow, B., 2024, ‘Preventing deformities in paediatric cerebral palsy in poorly resourced areas: A scoping review’, *South African Journal of Physiotherapy* 80(1), a2059. https://doi.org/10.4102/sajp.v80i1.2059

Note: Casting, serial casting – progressive stretch of a joint using casts to improve ROM; Spasticity, increase in muscle tone in response to a quick stretch, caused by central nervous system damage; Sustained Stretch, means 30 min or more in one position; Tilt table, standing frame with variable angle of inclination from vertical.

ADL, activities of daily living such as feeding, bathing; BMD, bone mineral density; CP, cerebral palsy; CPG, clinical practice guidelines; DF, dorsiflexion of the foot; ES, electrical stimulation; Ex, extension; GMFCS, Gross Motor Function Classification System; GM, Gross motor; HCP, health care professional; KAFO, knee-ankle-foot orthosis; LL, lower limb; MSK, musculoskeletal; ROM, range of motion of a joint; UL, upper limb; ROM, range of motion.

† , Hoffman reflex, test of spasticity, positive sign means increased tone and negative means no increased tone.

‡ , MAS, Modified Ashworth Scale: a grading system for muscle spasticity where 0 is normal and 4 is rigid.

There is overall weak and conflicting evidence for stretching interventions. Manual stretching interventions appear to be less effective for preventing deformities than sustained, positional stretching (Craig et al. 2016; Pin et al. 2006). Fragala et al. (2003) found that periods of non-intervention from a school-based manual stretch routine of 3 × 60 s per structure, taking place once to twice per week, produced inconsistent changes in LL ROM. The addition of ES to the antagonist during stretching significantly increased ROM and reduced spasticity (Groppe et al. 2012). Uzun Akkaya and Elbasan (2021) reported transient reduction in calf muscle spasticity after just 10 min of manual stretching, both with and without concurrent massage. No adverse effects were reported.

Wilton (2003), Autti-Rämö et al. (2006), Groppe et al. (2012) and Craig et al. (2016) all suggested that joint ROM may be maintained or increased in all limbs and spasticity reduced by an intensive serial casting programme if sustained for at least 3 weeks.

Positional stretching for 20 min using an orthotic in sitting or using a stander for 30 min at least three times per week for at least 8 weeks may be effective to increase LL ROM and may reduce calf muscle spasticity for up to 35 min after the stretch (Craig et al. 2016; Groppe et al. 2012; Laessker-Alkema & Eek 2016; Lannin et al. 2011; Pin et al. 2006). Wilton (2003) recommended wrist and hand orthotics be used at night or for 3–5 h per day to control deformities where spasticity is moderate to high although all other studies found inconclusive evidence of efficacy for ULs.

Although no stretching interventions resulted in improved motor function for the child, improved flexibility appeared to assist with symmetry in sitting (Physiopedia 2016) and ease of care when performing ADLs (Laessker-Alkema & Eek 2016). Adverse effects included hindrance of hand function while wearing UL orthotics (Autti-Rämö et al. 2006; Lannin et al. 2011; Wilton 2003) and bruising, skin breakdown, sleep disturbance, joint swelling and muscle cramps were reported in a few patients using LL orthoses (Craig et al. 2016; Laessker-Alkema & Eek 2016).

### Home-based intervention programmes

No studies were found evaluating the effectiveness of HBIPs to prevent MSK complications in severe CP. Twelve articles addressed the population of interest and aspects of HBIPs that influenced success.

Six were conducted in LMICs or involved RLSs. Three were reviews (Branjerdporn et al. 2021; Lord et al. 2018; Paleg 2005), and nine were primary studies, comprising eight qualitative studies (Bischof & Chirwa 2011; Colver et al. 2012; Halvarsson et al. 2010; Novak et al. 2012; Krüger & Sello 2008; Lillo-Navarro et al. 2015; Naidoo et al. 2019; Rezaie & Kendi 2020) and one quasi-experimental trial (Zuurmond et al. 2018). Studies explored essential programme components, training needs of caregivers and primary health workers, determinants of successful programme implementation and the impact on child development. Table 5 provides further details of each study.

**TABLE 5:** Home-based intervention programmes for cerebral palsy that include prevention of mitigate musculoskeletal complications: Content, impact and environmental factors.

Author, date, design and subject	Population	Methods and outcomes	Results and/or recommendations for HBIPs	Level, quality and rating
Paleg 2005 Narrative review *The role and content of early HBIP (HIC)*	Children with CP or at risk of CP	Elements of the home programme.	*Recommendations* - EI† should begin as early as possible at home. - Seating support from 4 months. - Supported standing from 9 months. - Treadmill training from 12 months if ambulation not expected.	Level VII 4/6 Moderate
Krüger and Sello 2008 Qualitative *Education status and training needs of rural parents of CP (LMIC)*	156 Parents of children with CP in rural South Africa	Survey and interviews: - demographics - social function of child - knowledge about CP - confidence to care for CP - programme needs.	*Caregiver characteristics* - 94% were mothers, 59% were unemployed and 83% had only 1° education. - 49% of children never accompanied parents on outings. - 87% had never received information about CP and 95% felt it was difficult to care for their child. - 100% wanted a HBIP, and 87% wanted a parent-teacher discussion. Teachers were ‘experts’. *Requirements for HBIP* - Appropriate to education and SES, easily implemented, inexpensive. - Should not oppose traditional beliefs. - Should incorporate basic brain structure, associated conditions and practical tips for care.	Level VI 6/10 Moderate
Halvarsson 2010 Qualitative *Parent experience of HBIP (HIC)*	10 Parents of children with CP, aged 3–19 years, GMFCS levels II-IV	Interviews: Parental role in HBIP (stretching programme), interaction with the child, time, coping strategies, views on professional support.	*Parental role in HBIP* - Programme evolved over time, ↑ participation of child in programme, parents went from authority to coach. - But programme may ↑ stress, ↑ demands on time. - Managed 30 min stretching maximum. - Coping with stress very important. *Views on HBIP* - Focus on mobility and activity. - Balance time with child versus family. - Adequate support from physiotherapists (knowledge, training in techniques, check-ins). - Evidence of effectiveness. - Stress management advice.	Level VI 8/10 Moderate
Bischof and Chirwa 2011 Qualitative *Prevalence of hip pain during ADLs in GMFCS levels IV and V (LMIC)*	13 children and young adults with CP, aged 8–26 years, non-ambulatory in a residential facility in South Africa	Observation of pain response during washing, dressing and transfers for 7 days.	*Pain prevalence* - During hip movement = 32.5%. - Pain ↑ if hips dislocated. - During washing was significantly ↑ than during dressing (*p* = 0.008) and transfers (*p* < 0.001). - Pain not always observed. - Handling techniques of caregivers affected pain levels and frequency.	Level VI 3/10 Low
Colver et al. 2012 Qualitative *Association between environment and participation in CP (HIC)*	818 children with CP aged 7–13 years	Home visit survey: Physical impairment and pain, participation in ADL, mobility, social interaction and recreation, school attendance, responsibilities.	*Home life outcomes* - Modified environment, hoists, (communication and mobility aids) → significantly positive influence on participation in meals, personal care and home life (all *p* ≤ 0.010). *Social life* - Adapted physical home environment, positive attitudes of family, friends and social support had significantly positive influence on child taking on responsibility (all *p* < 0.001) and relational participation (*p* ≤ 0.002). *Community life* - Positive teachers’, therapists’ and classmates’ attitudes had significantly positive influence on participation in school activities (*p* ≤ 0.02). - Positive attitudes of family, friends, social support led to significantly ↑ recreation participation (*p* ≤ 0.001).	Level VI 7/10 Moderate
Novak et al. 2012 Qualitative *Prediction of equipment needs per GMFCS level (HIC)*	242 Children with CP, aged 0–18 years, all GMFCS levels	Review medical records for equipment types and cost.	GMFCS level and presence of epilepsy predicted the prescription of assistive devices and technology (*p* = 0.000 and *p* = 0.008, respectively). Children in GMFCS levels IV and V had on average 10.00 pieces of equipment (s.d. 3.17).	Level VI 7/10 Moderate
Lillo-Navarro et al. 2015 Qualitative *Parent experiences of HBIPs and therapist teaching style (HIC)*	28 parents of children with disabilities	Focus group discussions. Characteristics and adherence to HBIPs’ perceived facilitators and barriers to adherence	*Characteristics of HBIPs* - Included both stretching and functional skill practice (locomotion, hand function, postural stabilisation). - 57% had 6–10 different exercises. - 54% did them daily. *Factors positively affecting adherence* - Fun. - Not painful. - Positive effects apparent. - Integrated into normal routines. - Not too time consuming. - Therapists equip, demonstrate, written instructions.	Level VI 8/10 Moderate
Zuurmond et al. 2018 Quasi-experimental *Impact of GTKCP programme ‡ for CP in Ghana (LMIC)*	75 caregivers of children with CP, aged 18 months–12 years, all GMFCS levels	Training with before and after interviews -parents’ quality of life and functional performance, worry, ADLs(mainly feeding), knowledge and confidence, child general health	*Quality of life* - Significant ↑ in median score from 12.5 to 51.4 (*p* < 0.001). *Knowledge and confidence* - Significant ↑ (*p* < 0.001). *Feeding and drinking* - Significant ↑ in median score from 29.7 to 51.6 (*p* < 0.001). *Perception about child’s general health* – Physical health → significant ↑ from 34% to 73% (*p* < 0.001), emotional health ↑ from 36% to 64% (*p* < 0.001).	Level III 6/9 Moderate
Lord et al. 2018 Systematic review *Determinants of effective HBIPs (HIC)*	17 sources, ≥ 145 parents of children with CP, aged 0–18 years, GMFCS all levels	- Relational aspects (therapist-parent and parent-child), - coping (feeling capable, supported) - priorities for interventions (training and resources)	*Priorities for HCP* - Build trusting relationships – be accessible and supportive, strengthen the parent-child bond. - Enable parent coping – feeling capable, finding support, coping strategies. - Prioritise the intervention – train, provide resources, tailor to the family, evidence based.	Level V 8/11 Moderate
Naidoo et al. 2019 Qualitative *CHW’s views on their competence and role for disabled children in rural SA (LMIC)*	32 CHWs and key informants in Ethekwini, KZN, employed for at least 1 year	Interviews and focus groups. - CHW’s knowledge and skills in childhood conditions. - barriers to service delivery. - training needs	*Competence* - 100% had exposure to disabled children but only 25% had had any specific training. - Felt unable to identify children and make referrals. *Barriers to service delivery* - ↓ transport, supervision, clear referral pathways. - Social stigma. *Training needs* - Health-specific training. - Better screening of children. - Standard-operating procedures and care pathways. - Knowledge about community resources.	Level VI 7/10 Moderate
Rezaie and Kendi 2020 Qualitative *OT’s views on factors influencing adherence to HBIPs for CP (LMIC)*	17 OTs from a metropolitan area in Iran, with at least 5 years’ clinical experience with CP	Interviews. - Child and family factors - Therapy and HCP factors	*Factors negatively affecting adherence* - Family – severity of disability, low SES, poor family support, disagreement on goals, poor experience of health services, lack of prioritisation of HBIP. - Therapist – low competence, communication, motivation, therapy – repetitive, long term, ‘meaningless’.	Level VI 6/10 Moderate
Branjerdporn et al. 2021 Systematic review *Comparison of parent-delivered vs HCP-delivered HBIPs in LMICs*	11 sources, 612 children with CP, aged 0–5 years	Child: - GM function, posture, positioning, feeding, self-care, social function Parent: - stress levels during feeding, positioning skills for feeding, feeding speed and support	Parent-delivered versus HCP-delivered interventions. *Child outcomes* - Small negative effect on GM function (mean difference – 0.41, 95% CI -5.31 to 4.49, *p* = 0.87) but small positive effect on posture, positioning and self-care (effect size 0.37, 95% CI -0.14 to 0.87, *p* = 0.16). - Small positive effect on social function (effect size 0.43, 95% CI -0.09 to 0.93, *p* = 0.10). *Parent outcomes.* - Significant ↓ in stress with training for exercise programme (odds ratio 1.67, 95% CI 0.51 to 5.40; *p* = 0.39) and during feeding (odds ratio 2.20, 95% CI 1.00 – 4.86; *p* = 0.05).	Level I 9/11 High

*Source:* Please see full reference list of Van Aswegen, S.R., Richards, M. & Morrow, B., 2024, ‘Preventing deformities in paediatric cerebral palsy in poorly resourced areas: A scoping review’, *South African Journal of Physiotherapy* 80(1), a2059. https://doi.org/10.4102/sajp.v80i1.2059

ADL, activities of daily living such as feeding and bathing; CHW, community health worker; CP, cerebral palsy; GM, gross motor; GMFCS, Gross Motor Function Classification System; HCP, health care professional, usually a physiotherapist or OT; HBIP, home-based intervention programme; HIC, high-income countries; LMIC, low- and middle-income countries; OT, occupational therapist; SES, socio-economic status.

† , EI, early intervention (children younger than 2 years).

‡ , GTKCP, ‘Getting to know cerebral palsy’, 11-month community-based training programme.

Three South African studies reported on caregiver and CHWs’ views on their capacity for identifying and managing CP in the community. Outcomes included knowledge of the condition, practical handling skills and pain caused during activities of daily living (ADL). A facility-based study found a moderately high prevalence of pain in children and young adults with severe CP during daily care activities, which they observed to be directly influenced by their handling techniques (Bischof & Chirwa 2011). Community healthcare workers required specific training in CP screening and clear referral pathways and protocols for further management (Naidoo et al. 2019). Caregivers desired better knowledge of the condition, practical training for everyday routines and ongoing involvement in support groups with health professionals and respected members of their communities (Krüger & Sello 2008).

Limited evidence suggests that, in spite of poverty and low education levels of caregivers, parent-implemented interventions in LMICs positively influenced posture, positioning, self-care, feeding and social function in children while reducing parental stress (Branjerdporn et al. 2021; Zuurmond et al. 2018), and that a positive, equipped and informed environment improved overall participation of children with CP at home, in school and the community (Colver et al. 2012).

Factors affecting fidelity and adherence to HBIPs by caregivers were the presence of trusting relationships between child, caregiver and therapist, and establishing skills and coping strategies (Lord et al. 2018). Priorities from caregivers included evidence-based interventions, the element of fun, the incorporation of the programme into daily routines and written instructions with demonstration (Lillo-Navarro et al. 2015). Caregivers prioritised programme flexibility and mobility goals for the child, as well as ongoing support for monitoring and programme adaptation over time (Halvarsson et al. 2010). Priorities from therapists included effective communication with families, joint goal setting and equipping in CP clinical skills and resources (Rezaie & Kendi 2020). Zuurmond et al. (2018) found that monthly telephonic or face-to-face check-ins with caregivers and a group chat via social media for therapists and/or CHWs were effective support mechanisms for their community-based programme.

## Discussion

The dearth of eligible high-quality empirical research in this review is notable, an observation also acknowledged in a recent overview of effective interventions for CP (Liguori et al. 2023). The ethical and methodological challenges of conducting primary research in this population were evident with limitations including non-randomisation, small sample sizes and low generalisability, hence, the few and broad nature of published guidelines that include prevention of MSK complications in severe CP. However, if pre-emptive strategies are not in place, we know from clinical experience that progressive MSK complications are common in CP and potentially devastating, which concurs with the recent findings of Tenaglia et al. (2022) and Krarup et al. (2024) who strongly advocate a comprehensive, targeted approach to limit MSK complications in children with CP. Although we found overall weak support for the use of 24-h PM for the prevention and control of hip displacement, scoliosis and musculoskeletal contractures (Chung et al. 2008; Damiano et al. 2021; Gericke 2006; Morgan et al. 2021; NICE 2012; Novak et al. 2020; Novak et al. 2020; Paleg et al. 2019; Pountney et al. 2002, 2009; WDHB 2014; Wynn & Wickham 2009), the importance of PM regimes in limiting deformities and promoting function is increasingly being recognised (Paleg & Livingstone 2022).

Considering that physical interventions aimed at limiting MSK complications require continuity and assimilation into everyday routines, being most effective when carried out daily or several times a week (Autti-Rämö et al. 2006; Craig et al. 2016; Gmelig Meyling et al. 2018; Gibson et al. 2009; Groppe et al. 2012; Laessker-Alkema & Eek 2016; Macias-Merlo et al. 2015, 2016; Martinsson & Himmelmann 2021; Paleg et al. 2013; Picciolini et al. 2016; Pin 2007; Pin et al. 2006; Tornberg & Lauruschkus 2020), it is reasonable to equip primary caregivers to perform them, where feasible, especially where formal health services are limited. Potential challenges in an RLSs would include the early identification of infants at risk so that interventions can be implemented in the home from as young as 4 months of age (Paleg 2005; Paleg & Livingstone 2022) and the availability and affordability of requisite equipment for lying, seating and standing, which is considerable for children in GMFCS levels IV and V (Novak et al. 2012).

Although efficacy of manual stretching techniques for preventing deformities has not been established, regular positional or sustained stretching regimes appear to maintain and improve ROM and temporarily reduce spasticity, which would likely facilitate positioning, daily care (e.g. perineal hygiene) and function (Craig et al. 2016; Groppe et al. 2012; Laessker-Alkema & Eek 2016; Pin et al. 2006). While serial casting and ES require an expert setting, manual and positional stretches would be feasible options for RLSs.

In RLSs, it is common to find relatively poor, less educated caregivers struggling to cope with the care of their child with CP, which they may experience as stressful and demanding. Often the perceived burden of care relates to difficulties with ADLs such as feeding and general lack of knowledge and confidence. We have shown that modifying the environment through parent-implemented approaches are acceptable and effective to improve child outcomes (Colver et al. 2012), ease of care and parental stress levels (Branjerdporn et al. 2021; Zuurmond et al., 2018), especially when accompanied by adequate training and support (Halvarsson et al. 2010; Lord et al. 2018; Naidoo et al. 2019). In RLSs, the inexperience and knowledge gaps of primary health workers in identifying families in need and supporting them may compound these issues. As these health workers are integral to successful implementation and sustainability, it is important to equip them with early identification tools, provide clear protocols for referral and train them in the HBIPs so they can better support families.

### Research gaps

Existing studies of PM have focussed on the role of sitting and standing programmes in prevention and control of the hip and LL deformities. Neuromuscular scoliosis is another serious, common complication of severe CP (Hagglund et al. 2018) that 24-h PM may influence; yet, supporting research is lacking. Studies on sleeping systems have been limited to commercially available options. The efficacy of low-cost equipment for lying positions should be a research priority, particularly in children classified as GMFCS levels IV and V who spend a significant portion of the day in lying.

Evidenced-based stretching protocols should be developed, including dosage and stretching modalities for different treatment goals. In our review, there were few reported adverse effects; however, some MSK discomfort during stretching might be expected. The contribution of any stretching to relieve or exacerbate pain is an important consideration and is likely to affect programme adherence. Other questions should be considered about the psycho-sensory aspects of touch during manual stretches and if it can be effectively combined with massage. These questions warrant further research.

Our review found very little research into content, efficacy of and adherence to HBIPs in RLSs that include physical modalities designed to prevent MSK complications in severe CP. Further research could help identify structural and social determinants of successful programmes, establish effective protocols and provide ways to quantify participation and functional outcomes.

### Strengths and limitations

This review charts the existing literature over the past 20 years on manual interventions for limiting MSK complications in severe CP, including general CPGs, and efficacy of individual modalities and home programmes. The existing knowledge and evidence gaps were highlighted to help prioritise future research for populations living in RLSs.

The search was broad, using multiple sources including online platforms and included quality rating to support any recommendations made. Using a non-categorical diagnostic approach might have yielded useful studies involving other diagnoses and increased our evidence base. Our limit on language may have excluded some relevant studies from LMICs but was necessary owing to limited translation services. Several of the retrieved articles were, however, originally published in other languages. Initial screening, extraction and quality appraisal by a single researcher could have resulted in selection bias, especially with such a broad scoping purpose. However, this risk was mitigated by establishing clear and detailed exclusion criteria *a priori* and bringing in a second, independent reviewer for full-text review. Measures were taken to improve intra-rater reliability.

## Conclusion

There is a paucity of high-level research and clinical recommendations for safe, feasible and effective home-based interventions to reduce MSK complications in conditionally or non-ambulatory children with severe CP living in resource-poor environments. This requires urgent redress if we are to meet the SDG-3 to ‘ensure healthy lives and promote well-being for *all* at *all ages*’ (UNDESA 2015), which would include *all* socio-geographic contexts.

In the meantime, we believe that if clinical reasoning and diligent monitoring are applied, the existing evidence for 24-h PM, stretching and splinting can be used to guide the development of a context-specific home-based programme to improve the outcomes for children with severe CP in RLSs.

## Footnotes

1 https://www.acpjournals.org/doi/full/10.7326/M18-0850?rfr_dat=cr_pub++0pubmed&url_ver=Z39.88-2003&rfr_id=ori%3Arid%3Acrossref.org.

2 https://app.covidence.org.

3 https://www.agreetrust.org/wp-content/uploads/2017/12/AGREE-II-Users-Manual-and-23-item-Instrument-2009-Update-2017.pdf.

4 https://jbi.global/critical-appraisal-tools.
